# The Role of Vitamin D in Inflammatory Bowel Disease: Mechanism to Management

**DOI:** 10.3390/nu11051019

**Published:** 2019-05-07

**Authors:** Jane Fletcher, Sheldon C. Cooper, Subrata Ghosh, Martin Hewison

**Affiliations:** 1 Nutrition Nurses, University Hospitals Birmingham NHS Trust, Queen Elizabeth Hospital Birmingham, Mindelsohn Way, Edgbaston, Birmingham B15 2TH 1, UK; 2 Gastroenterology Department, University Hospitals Birmingham NHS Trust, Queen Elizabeth Hospital Birmingham, Mindelsohn Way, Edgbaston, Birmingham B15 2WB 2, UK; sheldon.cooper@uhb.nhs.uk; 3 NIHR Biomedical Research Centre, University Hospitals Birmingham NHS Foundation Trust, Queen Elizabeth Hospital Birmingham, Mindelsohn Way, Edgbaston, Birmingham B15 2TH, UK; s.ghosh@bham.ac.uk; 4 Institute of Translational Medicine, University of Birmingham, Birmingham B15 2TH, UK; 5 Institute of Metabolism and Systems Research, The University of Birmingham, Birmingham B15 2TT, UK; m.hewison@bham.ac.uk

**Keywords:** vitamin D, IBD, Crohn’s disease, ulcerative colitis, supplementation, deficiency

## Abstract

Vitamin D has been linked to human health benefits that extend far beyond its established actions on calcium homeostasis and bone metabolism. One of the most well studied facets of extra-skeletal vitamin D is its activity as an immuno-modulator, in particular its potent anti-inflammatory effects. As a consequence, vitamin D deficiency has been associated with inflammatory diseases including inflammatory bowel disease (IBD). Low serum levels of the major circulating form of vitamin D, 25-hydroxyvitamin D (25-OH-D) are significantly more prevalent in patients with IBD, particularly in the winter and spring months when UV-induced synthesis of vitamin D is lower. Dietary malabsorption of vitamin D may also contribute to low serum 25(OH)D in IBD. The benefits of supplementation with vitamin D for IBD patients are still unclear, and improved vitamin D status may help to prevent the onset of IBD as well as ameliorating disease severity. Beneficial effects of vitamin D in IBD are supported by pre-clinical studies, notably with mouse models, where the active form of vitamin D, 1,25-dihydroxyvitamin D (1,25-(OH)2D) has been shown to regulate gastrointestinal microbiota function, and promote anti-inflammatory, tolerogenic immune responses. The current narrative review aims to summarise the different strands of data linking vitamin D and IBD, whilst also outlining the possible beneficial effects of vitamin D supplementation in managing IBD in humans.

## 1. Introduction

Inflammatory bowel diseases (IBD) are chronic, disabling diseases precipitating inflammation and ulceration throughout the gastro-intestinal tract (GIT). IBD is characterised by diarrhea, nocturnal defecation, abdominal pain, weight loss, and fatigue. The two principal forms of IBD are Crohn’s disease (CD) and ulcerative colitis (UC). Where UC usually only affects the colon, CD may affect the entire GIT from mouth to anus. IBD is common in developed, westernised countries with highest prevalence estimates of; UC 505 per 100,000 and CD 322 per 100,000 population in Europe and UC 249 per 100,000 and CD 319 per 100,000 population in North America [1]. The incidence of IBD is rapidly increasing in newly industrialised countries strongly implicating environmental factors [2]. The pathogenesis of IBD is not fully understood, but key influences are thought to include genetics, environmental factors, immune response, and gut microbiota [3]. The gut microbiota has emerged as an important feature in innate and adaptive immunity [4], with microbial colonisation of the gut being essential for the development of a mature immune system [5]. One explanation for the ongoing increase in IBD is the adverse effect of modern lifestyles on the composition and function of gut microbiota, including high saturated fat/high sugar diets and the use of antibiotics [6].

It is recognised that the availability of vitamin D is important in regulating gut mucosal immunity [3]; with studies suggesting that vitamin D may affect gut epithelial integrity, innate immune barrier function, and the development and function of T cells [7,8,9]. Although vitamin D deficiency is common in people with IBD it is not established if this is a cause or a consequence of the disease. However, there are suggestions that in genetically predisposed individuals, vitamin D deficiency may be a contributing factor in the development of IBD [7]. There is growing evidence that vitamin D status may affect disease activity. As such consideration should be given to screening and management of vitamin D deficiency in this patient group [10,11,12]. 

This narrative review explores the prevalence of vitamin D deficiency in people with IBD and the possible benefits to patients in treating this deficiency. Consideration is given to the management of vitamin D deficiency via exposure to sunlight, dietary sources and supplementation. 

## 2. Vitamin D Deficiency in IBD

### 2.1. Defining Vitamin D Deficiency

Vitamin D-deficiency is a common health issue across the globe, but it is especially prevalent in Northern European countries. Despite this, defining vitamin D deficiency in human populations remains a contentious issue. In 2010 the Institute of Medicine (IOM) defined vitamin deficiency as serum concentrations of 25-OH-D less than 20 ng/mL (50 nmol/L) [13]. Subsequently the Endocrine Society issued slightly different guidelines, defining vitamin D insufficiency as being serum 25-OH-D levels below 30 ng/mL (75 nmol/L) [14]. In the UK, the Scientific Advisory Committee on Nutrition (SACN) have taken a different approach [15], recommending that a serum 25-hydroxyvitamin D (25-OH-D) level of <25 nmol/L is the threshold at which vitamin D deficiency, and associated complications such as poor musculoskeletal health, is likely to develop. However, other guidelines and studies suggest that levels of 25-OH-D below 25–50 nmol/L constitute deficiency with levels >75 nmol/L indicating sufficiency [14,16,17,18,19]. The threshold between 51–74 nmol/L 25-OH-D may then be termed insufficiency. Crucially, these varying recommendations for vitamin D sufficiency and insufficiency continue to be based on the classical skeletal actions of vitamin D, and there are no current recommendations for more recently reported extra-skeletal actions of vitamin D and additional effects are associated with pharmacologic doses of vitamin D.

Both the Endocrine Society [14] and the National Osteoporosis Society [20] recommend that screening for vitamin D deficiency should be carried out in those at risk of vitamin D deficiency; they include people with IBD/malabsorptive disorders in the at risk group. Treatment for deficiency is recommended at a cut off of <50 nmol/L [14,20]. Nonetheless, current UK National Institute for Health and Care Excellence (NICE) and American College of Gastroenterology (ACG) guidance on the management of patients with CD and UC [21,22,23,24] do not address vitamin D deficiency in these patients.

### 2.2. Prevalence of Deficiency in IBD

Vitamin D deficiency is a public health concern with the NICE recommending oral supplementation in specific population groups including infants and children under 4 years, pregnant or breastfeeding women, people over 65 years of age and those who have low or no exposure to the sun. For example, those who cover their skin for cultural reasons, are housebound or confined indoors [25]. The population prevalence of vitamin D deficiency (serum 25-OH-D <40 nmol/L) in some westernised countries is reported to be between 30% [26,27] and 47% [28] with the highest levels reported for those people with darkly pigmented skin. However, people with IBD are likely to be at increased risk of developing vitamin D deficiency for a number of reasons including: impaired absorption of nutrients and bile salt malabsorption, restricted dietary intake, and medical advice to avoid/protect against sunlight exposure while taking immuno-suppressive treatments such as thiopurines [29]. 

A number of studies have evaluated the prevalence of vitamin D deficiency and insufficiency in people with IBD [30] (Table 1). Prevalence of vitamin D deficiency is highest during winter and spring and lowest during summer and autumn [31]. Where available reported prevalence in Table 1 corresponds with winter/spring levels. These studies suggest that vitamin D deficiency is generally higher in patients with CD than UC and usually higher than that of the general population. However, previous work by the authors suggest it is not standard clinical practice to measure vitamin D in these groups [30]. It is also important to recognise that whilst serum 25-OH-D deficiency has been widely reported for CD and UC, this may not necessarily reflect the status of other vitamin D metabolites. Notably analysis of the active form of vitamin D, 1,25-dihydroxyvitamin D (1,25-(OH)2D) has shown higher serum levels of this metabolite in patients with CD relative to UC patients [32]. The suggestion from this and other studies is that the increased inflammation in CD is associated with enhanced extra-renal conversion of 25-OH-D to 1,25-(OH)2D to support gastrointestinal anti-inflammatory immune responses. Measurement of serum levels of 1,25-(OH)2D is generally rare, but it is interesting to note that serum levels of this metabolite correlated inversely with bone mineral density in CD patients [32]. The functional impact of vitamin D on skeletal homeostasis in IBD is still unclear, and it is possible that link between elevated serum 1,25-(OH)2D and bone loss simply reflects the greater severity of inflammatory disease. Inflammation itself can drive osteopenia through pro-inflammatory cytokines [33]. Impaired vitamin D status has been reported to be a risk factor for low bone mineral density (BMD) in IBD patients in some studies [34], but not in others [35], and single nucleotide polymorphism variants of the gene for the vitamin D receptor (*VDR*) have been shown to protect against low BMD in patients with IBD [36]. Supplementation with vitamin D and calcium has been shown to improve BMD in pediatric [37] and adult [38] IBD patients. 

### 2.3. Vitamin D Deficiency in IBD—Cause or Consequence

Though vitamin D deficiency in IBD is well documented it is unclear if this is a cause or a consequence of the disease. It is notable that the highest prevalence of the disease appears in more temperate climates with lower levels of sunlight [8]. Some studies have suggested a correlation between vitamin D deficiency and increased disease activity [41,43,52,53,54]. Low vitamin D levels associated with IBD may be due to dietary restriction or low UV light exposure, but it is also important to recognise that genetic factors also contribute to variations in circulating vitamin D [55]. Single nucleotide polymorphisms (SNP) for components of the vitamin D system have been shown to contribute to the variation of serum 25-OH-D levels in IBD patients [56]. However, the contribution of SNPs to serum levels of 25-OH-D in IBD patients is very small (3%) [56], and subsequent Mendelian randomisation analyses have not shown any link between genetic determinants of vitamin D status and risk of IBD [57]. It is still unclear whether these observations support a causative relationship between vitamin D status and the development of IBD. A prospective cohort study of 72,719 women (age, 40–73 years) enrolled in the Nurses’ Health Study found that a higher predicted vitamin D status was associated with a reduced risk of developing CD [58]. However, it would seem that grounds for a causative relationship are most likely to be found in laboratory evidence of the functional effects of vitamin D, suggesting an effect of the active form of vitamin D on immune responses related to IBD [59].

## 3. Functional Effects of Vitamin D in IBD—Analysis of In Vitro and In Vivo Models

The mechanistic basis for a role for vitamin D in IBD stems firstly from studies of immuno-modulatory properties of 1,25-(OH)2D. These include antibacterial [60,61,62] and anti-inflammatory [63,64] actions on cell from the innate and adaptive immune system that modulate the pathology of gastrointestinal dysregulation and inflammation. Vitamin D also appears to play a pivotal role in the maintenance of gastrointestinal barrier integrity by regulating proteins associated with epithelial cell gap junctions [65,66,67]. The barrier function of vitamin D is also linked to its impact on the gastrointestinal microbiota, with serum 25-OH-D status in humans being correlated with changes in gastrointestinal bacterial genera associated with inflammatory immune responses [68,69]. In this way vitamin D has the potential to both prevent the onset of IBD via effects on barrier function and microbiota homeostasis, and also ameliorate disease progression through anti-inflammatory immune responses. 

**Figure 1:**
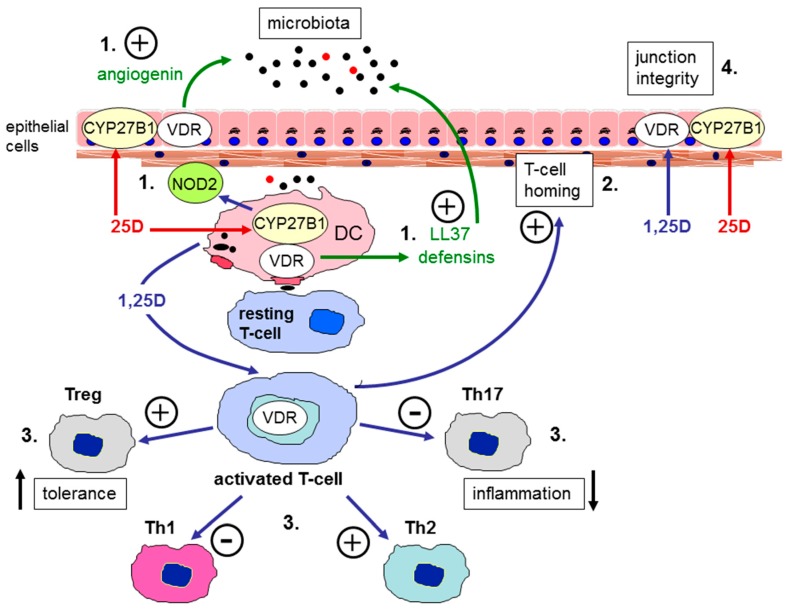
Vitamin D and barrier function in the gastrointestinal tract. Schematic representation of the expression of the vitamin D receptor (VDR) and vitamin D-activating enzyme (CYP27B1) in human colonic epithelial cells, antigen presenting cells such as dendritic cells (DC), and T cells. Immune responses to vitamin D occur either via systemic 1,25-dihydroxyvitamin D (1,25-(OH)2D) or local conversion of 25-hydroxyvitamin D (25-OH-D) to 1,25-(OH)2D. Possible target mechanisms include: 1) interface with microbiota (induction of antibacterials such as angiogenin, cathelcidin (LL37), defensins or intracellular pathogen recognition proteins such as nucleotide-binding oligomerisation domain containing 2 (NOD2)); 2) T cell homing to sites of inflammation; 3) suppression of inflammatory Th17 and Th1 cells and induction of tolerogenic Treg and Th2 cells; 4) enhanced expression of epithelial membrane junction proteins

The beneficial effects of vitamin D on gastrointestinal barrier function and immune surveillance are strongly supported by studies of mouse models that have incorporated dietary vitamin D restriction and genetic manipulation of the vitamin D metabolic and signaling system. Mice lacking the gene for the vitamin D receptor (VDR) that binds 1,25-(OH)2D show increased severity of experimentally-induced colitis that mimics IBD [70]. Similar observations have also been made for mice lacking the gene for 1α-hydroxylase, the enzyme that converts 25-OH-D to 1,25-(OH)2D [71]. Thus, in mice, inability to synthesise or recognise 1,25-(OH)2D was associated with increased IBD severity. This is due, in part, to impaired anti-inflammatory adaptive immune function [70], but has also been linked to disruption of the normal gastrointestinal epithelial barrier [66]. Interestingly, in mice with dietary vitamin D restriction increased colitis severity was associated with elevated levels of normal commensal bacteria within the gastrointestinal sub-mucosal epithelium, further underlining the importance of vitamin D in maintaining barrier integrity and surveillance of the gut microbiota [72]. The bioavailability of vitamin D for barrier and immunomodulatory activity within the gastrointestinal tract may also be influenced by the serum vitamin D binding protein (DBP) that transports vitamin D metabolites in the circulation. Although 25-OH-D binds with high affinity to DBP, it has been reported that the unbound or ‘free’ fraction of serum 25-OH-D is the most biologically active for immune responses to vitamin D [73]. Thus, the interaction between DBP and 25-OH-D may play a key role in gastrointestinal responses to vitamin D. For example, mice raised on diets containing exclusively vitamin D2 showed much lower serum levels of 25-OH-D compared to mice raised on vitamin D3 only. However, because 25-OH-D2 binds with lower affinity to DBP than 25-OH-D3, the levels of free 25-OH-D were similar in the two groups of mice and both showed similar patterns of experimentally induced colitis [74]. These observations suggest that free 25-OH-D may be a more sensitive marker of the protective effects of vitamin D on IBD. In addition to observations from mouse models, evidence from studies of humans also supports a link between vitamin D and IBD. This includes reports that 1,25-(OH)2D is a potent stimulator of nucleotide-binding oligomerisation domain containing 2 (NOD2), an intracellular pathogen-recognition receptor [75]. Mutations in the gene for NOD2 (CARD15) are associated with the development of CD [76,77], and vitamin D status may therefore act as a key environmental trigger for those individuals who are genetically predisposed to CD. Figure 1 is a schematic representation of the expression of VDR and the CYP27B1 gene (responsible for the production of 1α-hydroxylase) in human colonic epithelial cells and antigen presenting cells.

## 4. Managing Vitamin D Deficiency in IBD—Sources of Vitamin D

### 4.1. Sunlight Exposure

The main source of vitamin D in humans is via synthesis in the body stimulated by sunlight exposure [78]. The vitamin D precursor 7-dehydrocholesterol is produced by the liver and is stored in the skin. On exposure of the skin to ultraviolet B-light, this is converted to pre-vitamin D3 and enters the circulation. In the liver pre-vitamin D3 is converted to the inactive form 25(OH)D3. Renal conversion of 25(OH)D3 produces active form 1,25-(OH)2D3 [78]. 

Lack of adequate exposure to sunlight is likely to lead to vitamin D deficiency with levels <25 nmol/L [15]. However, it is difficult to determine what adequate exposure is. The British Dermatology Association in their 2010 consensus statement on the use of sun-exposure to prevent vitamin D deficiency noted the results of Rhodes et al [79]; the time to produce vitamin D is generally short and certainly before skin begins to burn or redden. The study found that sunlight exposure for an equivalent of 13 minutes of UK summer midday sun (approximate latitude 53.4808° N, 2.2426° W), three times per week over a six-week period was sufficient to increase serum vitamin D levels above 50 nmol/L. The study was carried out in Caucasian participants with one third of their skin exposed to simulated sunlight and wearing no sunscreen [79]. Darker skin required a longer exposure to sunlight to produce vitamin D, so, it would not be possible to have a single recommendation for all skin types. In addition, Rhodes et al note that excessive cutaneous exposure to sunlight may in fact lead to degradation of vitamin D but they do not quantify what excessive exposure is [79]. A more recent global consensus statement on the treatment and prevention of rickets suggests that the use of sunlight exposure to prevent or treat vitamin D deficiency is not feasible [80]. The authors note that there is no safe threshold of ultra violet exposure that allows for sufficient vitamin D synthesis without increasing the risk of skin cancer [80]. This is of particular relevance to patients with IBD, where there is an increased risk of non-melanoma skin cancer in those who have received thiopurines to treat IBD [81]. 

### 4.2. Dietary Sources

The majority of naturally occurring vitamin D in food is found in animal products [82] and, as in humans, the quantity of vitamin D found in animal products will vary according to the vitamin D status of the animal. This will be influenced by the animal’s diet, if they receive vitamin D supplementation in their feed and their exposure to sunlight [83]. In the USA the addition of vitamin D to livestock feed has been shown to improve the quality of the meat [84]. Despite the emphasis on animal sources of vitamin D, Black et al [85], stress the importance of further research in to edible plant-based foods in providing vitamin D in the diet. 

SACN [15] confirmed that the recommended daily intake for vitamin D from all sources (food and oral supplementation) is 400 IU per day for those aged 4 years and above. SACN estimate that this is a sufficient amount to maintain vitamin D levels >25 nmol/L. However, this remains below the levels recommended by the Endocrine Society and National Osteoporosis Society [14,20]. Nonetheless, maintaining this moderate intake from food sources alone is difficult where there are few foods containing significant quantities of vitamin D. 

NHS Choices [86] recommend the following sources of vitamin D:

- oily fish—such as herring, mackerel, salmon and sardines
- liver
- red meat
- egg yolks
- fortified foods—such as fat spreads and breakfast cereals

There are notable differences in the amount of vitamin D in each of these foods. Oily fish is a good source of vitamin D even though there are inter-source variations, such as differences between wild salmon and farmed salmon. However, oily fish may not be commonly consumed in some national diets. An analysis of UK Biobank dietary intake data showed that, of 164,573 sets of participant data, only 20% reported consuming two portions of oily fish per week [87]. The main dietary sources of vitamin D intake in UK adults aged 19–64 were; oily fish (11% of vitamin D intake), cereals (13%), eggs and egg dishes (13%), fortified fats (19%), and meat (providing 30% of total vitamin D intake) [15]. 

The UK National Diet and Nutrition Survey [88] reports that in adults aged 19–64 years, mean daily intake of vitamin D from dietary sources alone was 112 IU. When oral supplementation was included intakes increased to 356 IU in men and 136 IU in women, still below the modest recommended 400 IU/day.

#### Dietary Intake of Vitamin D in People with IBD

Studies have suggested that people with IBD may restrict their diet to help manage the symptoms of their disease. This has been shown particularly in people with CD and CD-related strictures [89] and even in remission diet may be sub-optimal. A cross sectional study was carried out with 67 CD patients in remission in Canada. Results showed that intake of vitamins C, D, niacin, thiamin, magnesium, phosphorus, potassium, and zinc were all significantly lower in CD patients compared to a control sample (*p* < 0.05) [90]. A recent UK cross sectional study of 67 patients with IBD (40 CD, 23 UC) found that 97% of patients reported food avoidance [91]. Commonly avoided foods were vegetables and wheat products. Mean vitamin D intake was 283 IU/day in the reported cohort. A further small cross-sectional [92] study of dietary intake in 31 patients with IBD in Iceland reported that patients avoided dairy (60%) and processed meat products (55%) to manage symptoms of their IBD. Some patients reported that fish had a positive effect on their symptoms (22%). A cross-sectional study carried out in the Netherlands [93] of 165 patients with IBD compared to healthy controls, demonstrated that patients with IBD consumed more meat and poultry with an average difference of 15.0g/day (95% CI 8.50–21.4); less dairy products at −36.3 g/day (95% CI −65.8–−6.84); and slightly less fish at −1.42 g/day (95% CI −0.94—−3.79). The findings of these studies would suggest that patients with IBD are unlikely to consume adequate amounts of vitamin D rich foods to achieve the moderate recommended intake. 

### 4.3. Vitamin D Supplementation

Oral supplements containing vitamin D are widely available both over the counter and in the form of prescription only medicines. Some may be single or multi-nutrient preparations. Supplements usually contain either ergocalciferol (vitamin D2) or cholecalciferol (vitamin D3); both forms of vitamin D are metabolised by the liver to 25(OH)D. However, studies suggest that vitamin D2 appears to yield less 25-OH-D than an equal amount of vitamin D3, with vitamin D3 being more effective at raising blood levels of 25-OH-D in humans [94,95]. For this reason, vitamin D3 supplementation is often recommended [14,20]. 

The dose of vitamin D supplementation required to prevent deficiency is an issue for debate. The European Food Safety Authority [96] has set an upper tolerable limit for vitamin D supplementation of 4,000 IU daily in adults. In line with the SACN report [15], current NICE [25] recommendations are 400 IU vitamin D daily. The IOM [13] suggest an intake of 600 IU daily to maintain a 25-OH-D level of >50 nmol/L, with the Endocrine Society [14] recommending a daily intake for adults of 1500 IU–2000IU. However, these guidelines do not address the risks for people with inflammatory, malabsorptive diseases such as IBD. A recent review has suggested that in IBD doses of 1800 IU–10,000 IU daily may be required [97]. With such a wide dosing range it is difficult to determine the amount that is likely to be most effective. 

## 5. Benefits of Treating Vitamin D Deficiency in IBD

It is widely accepted that vitamin D supplementation should be provided during corticosteroid treatment in IBD for prevention of deterioration of bone health, forming standard guidance. There is increasing interest in research investigating prescribing vitamin D supplementation for modulating inflammatory biochemical processed in IBD [97]. Oral supplementation of vitamin D has been shown to be safe with only minor side-effects which on the whole are generally tolerated among children, with no difference with dosage regimes such as 2000 IU/day versus 50,000 IU/week [98,99]. Adult studies show similar safety profiles [97], with meta-analysis confirming this with the most common side-effects including thirst, nausea, dry mouth, headaches, minor gastrointestinal upset, drowsiness, and fatigue [100,101].

Supplementation of 40,000IU of cholecalciferol weekly successfully significantly increased vitamin D levels in patients with active UC and among subjects with inactive UC and non-UC sufferers. Moreover, there was an associated significant reduction in inflammatory markers of colitis: both C-reactive protein (CRP) and fecal calprotectin [69]. This is not a finding in every study, however. A study from Korea whilst establishing a negative correlation between vitamin D levels and CRP in those with CD, supplementation had no impact upon CRP and disease indices; no association seen with vitamin D levels, CRP and disease indices in those with UC [102]. Whilst not meeting statistical significance (*p* = 0.06) relapse of CD was less common among subjects supplemented with 1200IU of vitamin D3, a group also showing an increase in serum vitamin D3 with supplementation [101]. 

With immuno-modulatory and biologic therapy increasing the incidence of infections, it is of interest that upper respiratory tract infections were less commonly observed in those supplemented, and indeed the greatest protective effect was observed among those with vitamin D deficiency provided with a modest 500 IU per day (versus placebo) [103]. 

Vitamin D also impacts upon hospitalisation and need for surgery. In an observational study of patients with both UC and CD, those with an insufficient serum vitamin D (25-OH-D) had an increased risk of requiring both hospitalisation and surgical intervention than those who were never deficient. This was independent of treatment strategies with immuno-modulators and anti-TNF alpha medication. This study measured vitamin D levels and different time points and identified those in whom the levels rose to normal, the majority of whom were taking supplements. This group significantly reduced their risk of requiring surgery with a drop in CRP also noted [12]. Whilst the study by Govani et al [104] did not examine vitamin D levels, they used the US National inpatient sample database, identifying patients with CD requiring surgery and established a UV exposure index based at 3-digit zip-code level. It was identified that those exposed to greater UV had a reduced risk of surgical intervention [104].

Vitamin D deficiency has been shown to be a risk for the development of colo-rectal cancer in people with IBD [105]. Whilst the study by Ananthakrishnan et al does not examine supplementation of vitamin D, their study of 2809 patients with vitamin D assay results (at least one) and development of cancer revealed that deficiency of vitamin D was associated with an increased risk of cancer: odds ratio 1.82 (95%CI 1.25–2.65), with an 8% reduction in risk for every 1 ng/mL increase in vitamin D level [105]. A recent randomised controlled trial on recurrence of adenoma in people without an inflammatory bowel disease showed less-promising results [106]. In the trial participants were randomised to receive either vitamin D, calcium, both agents, or placebo. No significant difference was found in any of the treatment regimens on the recurrence of adenoma. However, it is important to note that only participants with 25-OH-D levels >30 nmol/L <225 nmol/L were included [106]. Therefore, it can be argued that these results do not relate to a population who are vitamin D deficient such as those with IBD. Further research is warranted into the effects of treatment of vitamin D deficiency in IBD and the impact on the development of colo-rectal cancers in this group. 

IBD and anemia are linked, with the action of hepcidin implicated. Observational data has confirmed vitamin D deficiency is associated with increased hepcidin concentration and anemia [107]. This observational data has been strengthened with an interventional study revealing that even a short period of two-weeks of vitamin D supplementation was sufficient to not only increase serum vitamin D, but reduce serum CRP and hepcidin levels [108]. 

Vitamin D effects have been postulated to have an immuno-modulatory effect; a vitamin D analogue was shown to act as a TNF-alpha inhibitor of peripheral blood mononuclear cells, especially when used in conjunction with Infliximab, a TNF-alpha inhibitor [109]. Observation among 173 subjects receiving anti-TNF alpha medication showed those with a normal vitamin D level had an increased odds ratio 2.64 (95% CI 1.31–5.32, *p* = 0.0067) of having achieved remission at 3-months compared with those with low levels [110]. This finding is echoed in another retrospective observational study revealing that patients with IBD with an insufficient vitamin D level at initiation of anti-TNF alpha treatment were more likely to stop treatment due to loss of response (HR 3.49; 95%CI 1.34–9.09), again suggesting a beneficial effect of vitamin D repletion [111].

Meta-analysis of randomised control trials have been clear in identifying that supplementation, whilst on the whole was well tolerated and did increase serum vitamin D levels, were too heterogeneous in nature with regards to dosing and follow up-periods to draw any significant findings with improvements in serum inflammatory markers of CRP and ESR. There was however recognition that there were significantly fewer relapses in those supplemented with vitamin D (OR 0.34; 95%CI 0.20–0.58) but analysis by dosing did not yield further significant findings [100].

## 6. Conclusions

Vitamin D plays a significant role in the maintenance of gastrointestinal barrier integrity, surveillance of the gut microbiota and inflammatory immune responses. These mechanisms are important in both preventing the development of IBD and ameliorating symptoms of the disease. Nonetheless, vitamin D deficiency is common in people with IBD with prevalence being higher than the general population and somewhat higher in CD than UC [30]. The reasons for this are multi-factorial but include lack of sun exposure due to immuno-suppressive treatments, dietary restrictions [90], and impaired absorption of nutrients.

Whilst vitamin D deficiency is more common among those with IBD, data has been unable to establish if the relationship is causative or as a result of inflammation. Many serum markers of nutrition are affected by inflammation, making it challenging to understand cause or effect. However, through understanding of the mechanisms of action there is emerging evidence that vitamin D deficiency may be implicated in disease severity, if not in part the etiology of IBD.

There is a wealth of evidence revealing that intervention with oral vitamin D supplementation is safe and well tolerated. It has been more challenging to identify the advantages of restoring vitamin D levels in clinical disease progression, but more studies are beginning to reveal benefits. Aside from the well recognised skeletal effects, there is growing evidence for IBD disease outcome benefits from the normalisation of 25-OH-D serum levels in people with IBD. Benefits include: reduced risk of surgery in those with CD [12], reduction in inflammatory markers [69], reduction in the development of anemia [112], improved response to anti-TNF alpha treatment [111], and a reduction in the risk of colo-rectal cancer [105]. One study revealed a reduction in upper respiratory tract infections [91]. These benefits warrant attention to the vitamin D status of people with IBD. Several key questions remain to be answered. Firstly, it is still unclear what is the optimal circulating level(s) of 25-OH-D for prevention and/or management of IBD. Current guidelines such as the Institute of Medicine in North America [13], and Science Advisory Council on Nutrition in the UK [15] have been developed based on the regulation of calcium homeostasis and bone metabolism. As yet there are no similar guidelines for extra-skeletal effects of vitamin D. This is further complicated by the recent report of a human subject with homozygous deletion of the vitamin D binding protein (DBP) gene [113]. The woman in question had almost undetectable circulating levels of 25-OH-D but was nevertheless normocalcemic and had only had mild changes in bone metabolism, but had severe ankylosing spondylitis. This case report suggests that total serum measurement of 25-OH-D may not be the most accurate determinant of vitamin D ‘status’, but rather unbound or free 25-OH-D (which was relatively normal in the woman with the DBP gene deletion) is the driving force behind many actions of vitamin D [114]. This is likely to be an important consideration in future studies of vitamin D supplementation and clinical impact.

Maintaining or improving vitamin D intake by diet or increased sun exposure is problematic for people with IBD. Improvement in vitamin D status can be achieved by oral supplementation. There is however a lack of consistent outcome data for significant improvement in IBD outcomes, with variation in duration and dosing of supplementation, and many studies being retrospective in nature. The data surrounding enhanced response to biologic therapies with normalised vitamin D levels suggest that, with more interventional, double-blind, placebo controlled randomised trials, that individualisation of IBD management with supplementation of vitamin D in those who are deficient offers an important strategy in improving clinical outcomes.

## Figures and Tables

**Table 1:** Prevalence of vitamin D deficiency and insufficiency in inflammatory bowel disease (IBD) [30].

Study	*n*	Age Years Mean (SD)	Condition	25(OH)D <75nmol/L (%)	25(OH)D <50nmol/L (%)	25(OH)D <30nmol/L (%)
Bours et al [39]	185	50 (15)	UC		34	
	131	47 (15)	CD		44	
Caviezel et al [40]	57	41 (13)	UC		44	
	99	41 (14)	CD		58	
	25	48 (15)	IBS		28	
Frigstad et al [41]	178	39	UC		44	7
	230	40	CD		53	8
Gilman et al [42]	50	38 (10)	CD		44	6
Kabbani et al [43]	368	44 (10)	UC	29.9		
	597		CD	30		
Kuwabara et al [44]	41	39 (15)	UC		60	
	29	32 (7)	CD		100	
McCarthy et al [45]	32	37 (11)	CD		50	41
	32	37 (11)	HC		25	1
Pappa et al [46]	36	15 (3)	UC			25
	94	15 (4)	CD			38
Sentongo et al [47]	112	16 (4)	CD			16
Siffledeen et al [48]	242	40 (10)	CD		22	8
Suibhne et al [49]	81	36 (11)	CD	90	63	
	70	36 (9)	HC		51	
Ulitsky et al [50]	101	42	UC	67	46	
	403	43	CD	76	51	
Veit et al [51]	18	16 (2)	UC	83	50	28
	40	17 (2)	CD	73	40	15
	116	15 (2)	HC	75	27	10

UC = Ulcerative colitis; CD = Crohn’s disease; IBS = irritable bowel syndrome; HC = healthy control.
